# Assessing the efficacy of a stepped-care group treatment programme for borderline personality disorder: study protocol for a pragmatic trial

**DOI:** 10.1186/s13063-021-05327-0

**Published:** 2021-06-07

**Authors:** Judy A. Pickard, Adam Finch, Elizabeth Huxley, Michelle L. Townsend, Stephanie Deuchar, Kate L. Lewis, Jason Pratt, Brin F. S. Grenyer

**Affiliations:** 1 School of Psychology, University of Wollongong, Wollongong, Australia; 2 Illawarra Shoalhaven Local Health District, Wollongong, Australia

**Keywords:** Borderline personality disorder, Stepped care, Pragmatic trials

## Abstract

**Background:**

Borderline personality disorder (BPD) is a high prevalence and serious mental health disorder that has historically challenged the finite resources of health services. Despite empirical evidence supporting structured psychological therapy as the first line of treatment, there remains significant barriers in providing timely access to evidence-based treatment for this population. The primary aim of this study is to evaluate the effectiveness of providing a stepped-care structured psychological group treatment to individuals with BPD within local mental health services. The secondary aims of the study are to identify the variables that predict the need to step up or down in care and the effectiveness of treatment on psychosocial functioning.

**Methods:**

Participants seeking treatment at two community mental health services will be invited to participate. Randomised controlled trial assignment will be to either (i) group skills treatment or (ii) treatment as usual. Group treatment will be offered via a stepped-care pathway with participants initially attending a 12-week group with the option of a subsequent 16-week group. The criteria for inclusion in continuing treatment includes meeting > 4 BPD diagnostic criteria or severity on GAF (< 65) at the completion of the 12-week group. Data will be collected at baseline and at five follow-up time points over a 12-month period.

**Discussion:**

This pragmatic trial will provide valuable information regarding the effectiveness of a progressive stepped-care group treatment for individuals with BPD in the real-world setting of a community mental health service. It will further the current understanding of variables that predict treatment dose and duration.

**Trial registration:**

Australian New Zealand Clinical Trials Registry ACTRN12618000477224. Registered on 3 April 2018

## Background and rationale

Borderline personality disorder (BPD) is a high prevalence, serious mental health disorder that represents significant personal, social and economic cost [19]. People with BPD present frequently to outpatient mental health facilities and place a significant demand on hospital inpatient and emergency services [16]. Studies have reported as many as 20% of psychiatric outpatients, and over 30% of persons treated in an inpatient mental health unit have a diagnosis of BPD [2, 12, 14], with 11% of individuals also presenting with Axis I co-morbidity, e.g. mood disorder [33]. Guidelines for the treatment of BPD clearly stipulate the importance of structured psychological therapy in the community as the first line of treatment [23, 29]). Previous research has validated psychological therapies as effective in ameliorating the symptoms and the course of BPD, with several specific psychological therapies (dialectical behavioural or psychodynamic therapy) being equally efficacious and more effective than treatment as usual [6]. However, previous research is reported to be affected by issues with a heterogeneity of presentations, failure to find differences in treatment duration and frequency, and various sources of publication bias [6, 30]. The effects are small to moderate favouring BPD-tailored psychotherapeutic interventions when compared to non-specialised psychological interventions [6]. Modality of delivery has varied from individual therapy, group therapy or a combination. Limited health resources have generated an impetus for providing stand-alone group skills treatment in lieu of the standard dialectical behaviour therapy (DBT) model which combines both individual and group therapies. The findings provide overall support for the viability of stand-alone group treatment [18, 31], with a recent study conducted by Lyng et al. [20] in a community setting reporting no significant difference in the outcomes across the two treatment conditions. The authors proposed the findings support the feasibility of developing a stepped-care treatment approach for providing care to clients with varying risk profiles.

Empirically evaluated interventions to date are typically considered lengthy, e.g. 12-month programmes and long waitlists for these therapeutic options have resulted in poor access to timely care. Despite this, there is little evidence supporting the intensity of treatment, measured in hours and duration; Storebø et al. [30] states: “We compared the effects of less than six months versus six to 12 months versus above 12 months duration for the outcome of BPD symptom severity. We found no evidence of significant differences between the subgroups” p. 65.

Further, DSM V criteria require that individuals satisfy only five of the nine diagnostic criteria to meet diagnosis; in effect, this results in 256 possible combinations or presentations of the disorder [19]. Given the subsequent heterogeneity of presentation, a “one-size-fits-all” treatment approach is arguably not sufficient for this population. A recent review of treatment for BPD found five psychotherapeutic interventions equally efficacious and with notable commonality in approach [15]. The authors proposed that the availability of more than one type of therapeutic intervention would better address issues with heterogeneity, patient preferences and finite resources. Further, Huxley et al. [10] reported on the effectiveness of a brief 3–4 session intervention as an initial step in the treatment journey. The authors noted that the variety of pathways patients followed subsequent to the intervention was reflective of the heterogeneity of crisis presentation, supporting the importance of variety in intensity and treatment type for this population. The need for further research investigating the effectiveness of modified structures in service provision, e.g. stepped care, and the identification of pre-treatment variables, e.g. diagnostic criteria to guide treatment choice and intensity, is clearly evident.

## Stepped care

Psychological therapy is considered as a primary intervention for people with BPD (National [23]); however, the finite resources of mental health services are challenged by client need and have historically fallen short in providing timely access to appropriate care. A recent review of care for people with BPD by Lawn and McMahon [13] noted that 52.5% of respondents (n = 105) reported difficulty in accessing care, attributing this to lengthy waitlists, financial barriers, physical distance to care and dismissing attitudes from health staff. Implementing stepped-care psychological therapy models compared to treatment as usual has been shown in a randomised controlled trial to reduce demand for hospital services, including re-presentations to hospital, shorter bed days and thus significantly reducing costs [8]. A follow-up study demonstrated how the stepped-care psychological approach retained people in care and reduced BPD symptoms and increased quality of life [10].

Stepped-care approaches, which are more responsive to client need, provide an alternative to fixed longer-term models that have high dropout rates [15]. The National Institute for Health and Clinical Excellence [25] guidelines support the adoption of stepped-care models to better meet the needs of clients with mental health problems. Stepped care is designed to more adequately address patient needs by improving accessibility to care and adjusting treatment intensity in health care settings with limited resources [25]. Stepped-care treatment is underpinned by two principles. Firstly, treatment provided is “least restrictive”, and secondly, it is “self-correcting”. In the context of public health services, these principles are applied by orientating stepped-care models towards providing treatment access to the minimal required specialist treatment (*least restrictive*), with the flexibility to step up or down (*self-correcting*) as warranted and based on individually tailored clinical decision-making, e.g. increase in acuity, poor response to care [3]. Stepped-care models are implemented as either progressive or stratified. A progressive model is one where the individual begins treatment with minimal intensity, e.g. brief or internet-based and progresses through a line of treatment options with increasing intensity in regard to clinician contact and format (group vs individual). A stratified model relies on individual assessment to gauge client need and enters them into the treatment phase most appropriate to clinical presentation [26]. To date, there has been limited research exploring the variables that may predict stepping up or down in care, despite its clear importance for ensuring the effective implementation of the stepped-care approach.

## Objectives

The proposed study aims to evaluate the effectiveness of a stepped-care structured psychological intervention for BPD when compared with treatment as usual. Specifically, the research seeks to answer: Is a stepped approach effective in treating BPD? This will be answered through the following questions.

(i) Is there a difference in outcomes for clients receiving group intervention or treatment as usual?

Hypothesis 1. Patients in the group treatment will demonstrate an equivalent reduction in BPD symptoms to participants in the treatment as usual (TAU) condition.

(ii) Do changes in symptoms and functioning during treatment persist following treatment?

Hypothesis 2. Participants in the group treatment will report an equivalent reduction in BPD symptom severity to participants in the TAU condition following treatment.

(iii) What implications does a stepped-care approach have for health services (including economic, treatment focus)?

Hypothesis 3. Participants in the group treatment will experience an equivalent reduction in length of admission and acute care following treatment compared to participants in the TAU condition.

This study is being conducted within two community mental health services which positions it as a pragmatic trial designed to provide valuable information regarding the real-world effectiveness of stepped-care treatment for BPD compared to existing care (treatment as usual).

## Research design and methods

### Study design

**Fig. 1:**
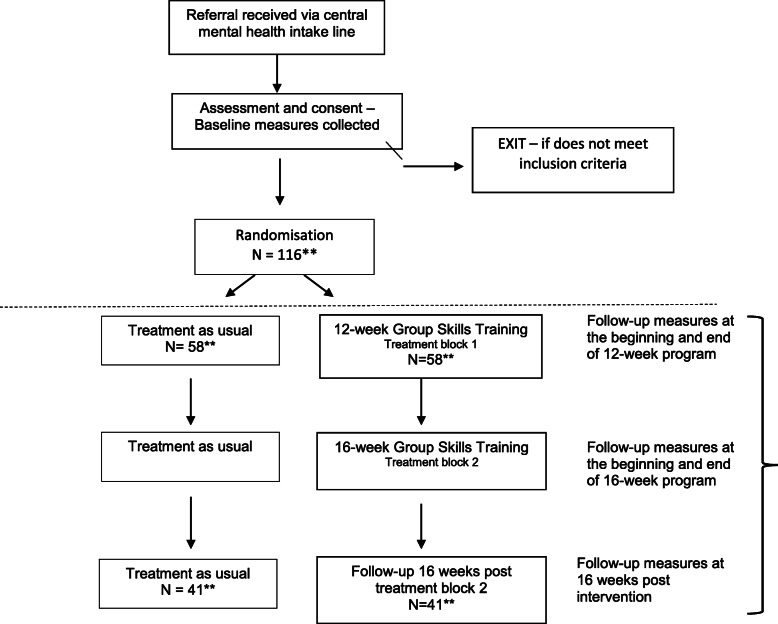
Proposed trial design evaluating stepped group skills training interventions. Note. *All participants will be contacted 16 weeks post-intervention, independent of the number of sessions completed. **Number of participants expected at each time point (inclusive of a dropout rate of 30%)

The current study is a multi-centre, prospective, randomised, non-blind, superiority parallel arm trial comparing a stepped-care model of stand-alone DBT group skills therapy with treatment as usual. The subject of this evaluation is a two-step care model that was developed to address the finite resources within local community mental health services (see Fig. 1 for the trial design flow chart). Referrals for treatment will be made through a central mental health intake line or emergency department. All participants will complete a diagnostic assessment to determine suitability for the study. Following recruitment, participants will be randomly allocated to one of the two conditions. The treatment condition (group skills therapy) will be a manualised DBT programme.

The group skills treatment will involve two steps. The treatment group will be offered a weekly, 2-h group treatment for 12 weeks. Following completion of the 12-week group, suitability for inclusion in the second step of group treatment (16-week skills group) will be assessed based on group attendance and clinical indicators: criteria for inclusion in continuing treatment includes meeting > 4 BPD diagnostic criteria or severity on GAF (< 65) at the completion of the 12-week group. Similarly, participants in the TAU condition will be retained in treatment through the additional 16-week period based on clinical need.

DBT targets the development of skills in mindfulness, emotional regulation, distress tolerance and interpersonal effectiveness [17]. Two separate group treatment manuals will be developed for the treatment condition. The manualised treatments will be based on the DBT skills and provide structured therapeutic intervention. The 12-week manual will provide an introduction to target skills and an opportunity to practise within the group. Participants will be encouraged to practise skills between sessions and provide feedback in the following session. The 16-week manual will expand on skills introduced in the 12-week manual. It will be delivered in a similar format, including in-session and between-session practice with a review.

Treatment as usual (TAU) refers to typical care provided by the trial sites. Participants randomised to this condition will be referred to the care pathways typically used by the public mental health site. These referrals will be informed by clinical decision-making and the presenting issues of the participant. As such, a range of treatment settings may be accessed in the TAU condition including case management within the local community mental health service, mental health rehabilitation services, referral to a local private psychologist or psychiatrist with known experience in working with BPD, local non-government organisations, general practitioners (GPs) and family and carer services. The TAU referral pathway will be specifically tailored to the participant presentation.

### Study setting

Treatment will be provided at two local community mental health sites. They are as follows: (1) the Illawarra Community Mental Health Service, which is located in Wollongong, NSW, Australia. Wollongong is a coastal regional city with an estimated population of 216,071 [1]; (2) the Campbelltown Community Mental Health Service is located in Campbelltown, NSW, Australia. Campbelltown is located in the south-western suburbs of Sydney, NSW, and has an estimated population of 170,943 [4]. The median income levels for both sites are reportedly below the Australian median income level. Approximately 80% of participants will be recruited at trial site 1 with the remaining 20% recruited at trial site 2.

This study uses a collaborative multi-method approach between NSW Health clinicians and clinical researchers at the University of Wollongong to ensure the outcomes will have an application to clinical research and practice and broader service delivery [11]. The protocol is in accord with the Standard Protocol Items Recommendations for Intervention Trials (SPIRIT) guidelines (see Table 1).

**Table 1:** SPIRIT table

Domain	Measure	Study period						
		Recruitment	Baseline	Randomisation	Intervention			Follow-up
Week		**0**	**0**	**0**	**4/16**	**16/36**	**4/36**	**52**
**Contacted by**		**CP**	**CP**	**CP**	CP	**CP/RA**	**CP/RA**	**RA**
**Enrolment**								
Eligibility screen		**X**						
Informed consent								
Diagnostic assessment			**X**					
Allocation			**X**					
**Intervention**								
Group skills programme (12 weeks)					Weeks 4–16			
Group skills programme (16 weeks)					Weeks 20–36			
Treatment as usual					Weeks 4–36			
**Assessments**								
Diagnostics	SCID-5-CV		**X**					
	SCID-5-PD		**X**					
Personality traits	PID-5-BF		**X**					**X**
	SA-SAPAS		**X**					**X**
	MSI-BPD			**X**	**X**	**X**		**X**
Executive functioning	BRIEF-A		**X**					**X**
Mental health	K-10		**X**		**X**	**X**		
	MHI-5			**X**	**X**	**X**		**X**
	FFMQ-SF			**X**	**X**	**X**		
	ISAS			**X**	**X**	**X**		
	DERS			**X**	**X**	**X**		
	BDI suicidal ideation item			**X**	**X**	**X**		**X**
	Self-harm (developed items)			**X**	**X**	**X**		**X**
	DEQ-SC6			**X**	**X**	**X**		**X**
	Single item based on the DES			**X**	**X**	**X**		**X**
Quality of life and psychosocial functioning	GAF		**X**					**X**
	SOFAS		**X**					**X**
	HONOS		**X**		**X**	**X**		
	APQ-6		**X**		**X**	**X**		
	WHO-QOL BREF items			**X**	**X**	**X**		**X**
	WHO-DAS items			**X**	**X**	**X**		**X**
	Global Health item			**X**	**X**	**X**		**X**
Relationships and attachment	Support person developed items			**X**	**X**	**X**		**X**
	RQ			**X**	**X**	**X**		**X**
	ORS						**X**	
Therapeutic alliance and engagement	Engagement in treatment (developed items)					**X**		
	Treatment satisfaction (developed items)					**X**		
	Penn Helping Alliance Questionnaire					**X**		
	SRS						**X**	
Biological measures	Saliva samples				**X**	**X**	X	

*Note*: X—administered at time point

*SCID-5-CV* Structured Clinical Interview for DSM-5 Disorders, *SCID-5-PD* Structured Clinical Interview for DSM-5 Disorders for Personality Disorders, *PID-5-BF* Personality inventory for DSM-5 – Brief Form, *SA-SAPAS* Self-Administered Standardised Assessment of Personality – Abbreviated Scale, *MSI-BPD* McLean Screening Instrument for Borderline Personality Disorder, *BRIEF-A* Behaviour Rating Inventory of Executive Function – Adult Version, *K10* Kessler Psychological Distress Scale, *MHI-5* Mental Health Inventory 5, Five Facets of Mindfulness-Short Form, *ISAS*Inventory of Statements about Self-Injury – Section II. Functions, *DERS* Difficulties in Emotion Regulation Scale, *BDI* Beck Depression Inventory, *DEQ-SC6* Depressive Experiences Questionnaire Self-criticism subscale, *GAF* Global Assessment of Functioning Scale, *SOFAS* Social and Occupational Functioning Assessment Scale, *HONOS* Health of the nation outcome scale, *APQ6* Activity and Participation Questionnaire, *WHO-QOL* World Health Organization Quality of Life BREF, *WHO-DAS* World Health Organization Disability Assessment Scale, *RQ* Relationship Questionnaire Clinical Version, *ORS* Outcome Rating Scale, *SRS* Session Rating Scale, *CP* clinical psychologist, *RA* research assistant

### Participants

Participants referred through the central mental health intake line for treatment for emotional dysregulation, self-harm and suicidal ideation and behaviour will be offered a diagnostic assessment including a semi-structured interview (SCID-5-CV and SCID–5-PD) to confirm the diagnosis of borderline personality disorder. This will be conducted by a clinical psychologist or registered psychologist.

Inclusion criterion: Current diagnosis of BPD—DSM V criteria.

All participants will be 18 years or over due to the trial sites being adult mental health services.

Exclusion criteria: Potential participants will be excluded if they meet the following criteria: (1) unlikely to be able to engage with the intervention (e.g. due to geographical constraints, severe substance use or current mental health symptoms which impair their ability to participate in therapy) and (2) assessed to pose a significant risk to the participant (i.e. they require a higher degree of care than can be provided within the trial).

### Sample size and power analysis

A clinically meaningful difference was defined as a reduction of 16% or more in the number of BPD symptoms, according to the MSI-BPD. This estimate is informed by previous studies using this measure, clinical judgement and existing trials examining shortened skills-based interventions for BPD [21, 22]. For this trial, the difference to be detected was calculated using the mean baseline MSI-BPD score (8.16) and standard deviation (2.08) in Miller et al. [22]. The reduction of 16% represents the within-subject mean symptom reduction at the end of the 16-week treatment block, relative to the individual’s baseline. Thus, based on a power of 80%, an alpha level of .05, the total number of participants required in each group is 40. However, accounting for a drop-out rate of 30%, 58 participants will need to be recruited for each group (N = 116).

### Participant randomisation

Following diagnostic assessment, participants will be randomly allocated to one of the treatment arms using a computerised randomisation programme, QMinim. QMinim uses a minimization method to generate an imbalance score for each participant based on prognostic factors. Allocation is then made to ensure minimum imbalance between the groups. Randomness is incorporated into the generated algorithm to maintain blindness [28]. Blocking is used to ensure equal numbers within each condition. Participants will be allocated to each treatment arm on a 1:1 ratio. Minimization prognostic factors are age, gender, GAF score severity, self-harm ideation and suicide attempts to control for these variables and allow for balanced groups. Treatment allocation will be concealed from the researchers administering assessments to prospective participants to ensure bias cannot be introduced into the allocation process.

### Baseline and follow-up evaluation

The McLean Screening Instrument for Borderline Personality Disorder ((MSI-BPD) [32] and the SCID-5-PD will be conducted at baseline for diagnosis. Evaluation will take place at the completion of the 12-week and 16-week interventions and 16 weeks following the end of treatment to assess the change in BPD symptom using the MSI-BPD. All participants will be contacted for assessment at these time points regardless of treatment arm or continuation in treatment.

#### Symptom and psychosocial evaluation

Baseline measures administered at the assessment will include widely used valid and reliable measures of symptoms including BPD symptomatology, depression, disability and global functioning (see Table 1). These measures will be re-administered at the beginning and end of each block of treatment in both conditions. They will then be administered at 16-week follow-up interviews conducted following the end of treatment.

#### Service utilisation evaluation

Service utilisation data for 16 weeks prior to recruitment with this study and 16-week post-treatment follow-up will be collected for both groups regardless of the number of sessions attended. This will include presentations to the emergency department and inpatient settings, and length of engagement in these acute services.

### Data management and linkage

Data will be managed according to the principles outlined in the NHMRC’s Australian Code for the Responsible Conduct of Research [24]. Specifically, the original data will be stored in hard copy and electronic form. Data will be entered into a database with no identifiable information other than a unique participant identification code. Electronic data will be encrypted and stored on a restricted access shared drive. De-identification of data will be managed by the 4th author. The research team will have access to the de-identified information on the shared drive for data analysis. Regular audits of data for data entry reliability and security will be conducted*.* A trial steering committee will be established with responsibility for overall research design, planning and implementation. Membership of the steering committee will include the chief investigator and representatives from all stakeholders, e.g. mental health service.

As part of this study, participants’ trial data will be linked with mental health service admission information from their electronic medical records.

Regular audits of the trial protocol will be provided to the funding body. This will include information regarding research design, implementation and participant recruitment and dropout. Alterations to the research protocol will be updated in the clinical trial registry (ANZCTR). Any adverse events will be reported to the Human Research Ethics Committee.

### Primary outcomes

Two primary outcomes will be assessed in this study: symptom reduction and service utilisation. Specifically, the reduction in BPD symptom severity and associated mental health symptoms will be measured by the MSI-BPD total score [32], and the use of health services including emergency department visits and inpatient admissions will be assessed to evaluate the efficacy of the two treatment conditions.

### Data analysis plan

#### Characteristics of the sample

Descriptive statistics will be used to summarise the clinical and demographic characteristics of the sample at baseline.

#### Primary analyses

The effect of treatment on primary outcomes will be evaluated using a linear mixed models approach, to account for the repeated measured structure of the data (SPSS-25). The data will be analysed as an intention-to-treat with time as a repeated measure. Recommended as the gold standard for study designs of this nature, this design (i) allows for the assessment of individual trends over time, with sensitivity to detect change; (ii) takes into account the longitudinal nature, utilising all data available; and (iii) controls for any pre-intervention differences [9, 27]. A linear mixed models analysis will be performed for each primary outcome variable symptom reduction (total number of BPD symptoms measured by the MSI-BPD) and service utilisation (inpatient hospital admissions and emergency department presentations). Change over time on these variables will be modelled as a within-subject effect of time, and the effectiveness of the intervention conditions will be determined by significant two-way group × time interaction. Although the change between baseline and end of the 16-week treatment block is of primary interest, three time points will be included to also examine the change during treatment and whether any changes are maintained following treatment. Within- and between-group effect sizes will be calculated, compared and interpreted according to the Cohen [5] recommendations.

#### Economic analyses

An important component of this study will be to examine and compare the cost-effectiveness and efficacy of the stepped individual and skills group interventions. Using a pragmatic clinical trial design, this study will include the routine costs and healthcare service utilisation costs associated with the implementation of both interventions. Service utilisation data, e.g. the number of presentations and admissions will be used to compare healthcare service costs between the two treatment groups. Although healthcare costs are measured in monetary terms, clinical effectiveness is determined independently in terms of clinical outcomes. A cost-effectiveness analysis will be performed [7].

### Planned dissemination of trial results

Trial results will be made available through publication in peer-reviewed journals, conference presentations and health service reports.

## Discussion

There has been a significant increase in knowledge regarding the effectiveness of psychological interventions in the treatment of BPD over the past 20 years, and it is now commonly held that structured psychological treatment is the primary treatment approach ([15, 23]). Despite this, health services are overwhelmed by the demand for services and struggle to provide timely access to treatment. The proposed stepped-care model provides a progressive treatment pathway for our evaluation. Findings from this study will help inform community mental health services in their mission to provide timely access to effective treatment with limited resources. Furthermore, data collected at baseline regarding symptomatology will assist our understanding of the impact of heterogeneity on treatment outcomes. It is hoped that this will help progress the development of diagnostic profiles for guiding treatment choice. The findings of this study will also provide important information about treatment dosage for BPD, and factors which may influence the need for greater or less intensive treatment.

There are several limitations in this study which relate primarily to it being conducted within two busy and active community mental health services that experience the problem of limited resources. Firstly, the inclusion of a treatment arm receiving the standard 12-month DBT treatment protocol could potentially provide greater confidence in significant treatment effects for the stepped-care model; however, this was not possible due to resource limitations. Secondly, we are unable to control the interventions provided to the TAU group. Consequently, the TAU group could potentially receive interventions that differ in intensity, therapeutic approach and mode of delivery, which therefore may affect the outcomes. Treatment as usual may also include referral to a number of services within the community. Although the follow-up survey will attempt to ascertain information regarding intensity and type of therapeutic approach, the accuracy of this information may vary.

### Trial status

The trial is registered with the Australian New Zealand Clinical Trials Registry - ACTRN12618000477224. It was first registered prospectively on 3/04/2018 and updated on 11/03/2020. TRGS protocol version 1, date of protocol: 17/08/2017. Data recruitment commenced on 5/10/2018. To date, 125 participants have been recruited to the study. It is anticipated that recruitment will be completed by October 2020.

## Data Availability

The datasets used during the current study are available from the corresponding author on reasonable request.
